# Boosting antibody responses to *Plasmodium falciparum* merozoite antigens in children with highly seasonal exposure to infection

**DOI:** 10.1111/j.1365-3024.2009.01193.x

**Published:** 2010-04

**Authors:** O J AKPOGHENETA, S DUNYO, M PINDER, D J CONWAY

**Affiliations:** 1 Medical Research Council LaboratoriesFajara, The Gambia; 2 Department of Infectious and Tropical Diseases, London School of Hygiene and Tropical MedicineLondon, UK

**Keywords:** malaria, IgG, immunological memory, antibody avidity, malarial vaccine, merozoite antigens

## Abstract

*Longitudinal cohort studies are important to describe the dynamics of naturally acquired antibody response profiles to defined* Plasmodium falciparum *malaria antigens relative to clinical malaria episodes. In children under 7 years of age in The Gambia, serum IgG responses were measured to* P. falciparum merozoite *antigens AMA1, EBA175, MSP1*_*19*_*, MSP2 and crude schizont extract, over a 10-month period. Persistence of antibody responses was measured in 152 children during the dry season when there was virtually no malaria transmission, and 103 children were monitored for new episodes of clinical malaria during the subsequent wet season when transmission occurred. Children who experienced clinical malaria had lower antibody levels at the start of the study than those who remained free from malaria. Associations between dry season antibody persistence and subsequent wet season antibody levels suggested robust immunological memory responses. Mean antibody levels to all antigens were elevated by the end of the wet season in children who experienced clinical malaria; each of these children had a boosted antibody response to at least one antigen. In all children, antibody avidities were lower against MSP2 than other antigens, a difference that did not change throughout the study period or in relation to clinical malaria episodes.*

## Introduction

*Plasmodium falciparum* infection remains a significant cause of mortality among children in malaria endemic countries. High antibody levels are generally associated with subsequent reduced susceptibility to clinical malaria (1–6). Antibody levels usually increase markedly within 1 or 2 weeks after the onset of symptoms when individuals who have previously had malaria are exposed to a new malaria infection (7–9). Although antibody levels normally decline after an infection is resolved, the persistence of moderate antibody levels is seen in a substantial proportion of individuals, and this persistence has been shown to increase with age among young children (10). Such persistent antibody production might be a marker of humoral immune memory capacity, but it is unknown whether this correlates with the ability to make rapid boosted antibody responses upon exposure to subsequent *P. falciparum* infections. Studies of serum antibodies prior to, during, and following clinical *P. falciparum* infections are important to investigate this.

It has been proposed that recent episodes of clinical malaria in humans are associated with lower antibody avidities in comparison with individuals who have resolved clinical infections, suggesting that higher antibody avidity is associated with enhanced immunity to clinical infection (11–13). Antibody avidity maturation was described in a murine model of *Plasmodium chabaudi* infection, indicating increasing antibody avidities after primary and secondary infections, and continued increase after multiple infections (14). One study suggests that increases in antibody avidities occur during resolution of clinical *P. falciparum* infection in humans, reflecting maturation of antibody responses to *P. falciparum* (11), and another indicates that high avidity antibodies are associated with protection from clinical malaria (15).

This study takes advantage of the highly seasonal pattern of malaria transmission in The Gambia, where *P. falciparum* transmission is very low during the annual dry season and high during the wet season, which leads to highly seasonal incidence of clinical malaria. Naturally acquired antibodies were examined at different time points during a dry and subsequent wet season. The study cohort of children up to 7 years of age in The Gambia was previously analysed for the determinants of persistence of antibody levels to several merozoite stage antigens during the dry season (10). Here, antibody responses during the wet season were analysed, including assay of antibody avidity. The antibody response profiles were examined in relation to individual episodes of clinical malaria, and for the cohort as a whole over time including the preceding dry season.

## Materials and methods

### Study area

In The Gambia, transmission of malaria occurs between July and December, during and immediately following the annual rainy season, with peak incidence of clinical malaria normally between September and November (16). All study subjects were living in The Gambia in the town of Farafenni and surrounding villages. The studies were reviewed and approved by the Medical Research Council Scientific Coordinating Committee and the Medical Research Council and Gambian Government Joint Ethics Committee.

### Dry and wet season cohort (February–December 2004)

In February and March 2004, parents or caregivers of 152 children under 74 months of age gave informed consent for a community-based survey of infection and antibody levels over a 12-week period. From each child, a venous blood sample (∼5 mL) was collected at enrolment (day 0), for serum and thick blood smear preparation, and finger prick (∼300 μL) blood samples were requested every 2 weeks.

At the end of the 12-week dry season study, all participants were provided with bed-nets and deltamethrin insecticide treatment for use throughout the subsequent wet season. Approximately 1 month later, during the wet season, written informed consent was sought for participation in a follow-up study of malaria and antibody responses. A finger prick sample for serum and thick blood smear preparation was collected early during the wet season in July and at the end of the wet season in December. Children were visited by field workers every week between these two surveys, and if they had fever (axillary temperature ≥37·5°C) or reported history of fever within the previous 48 h, a finger prick blood sample was taken and tested immediately for malaria using a rapid diagnostic test (RDT – OptiMAL®; DiaMed, Dakar, Senegal). A thick blood smear was taken for confirmatory laboratory analysis, and the remaining volume was collected for serum. If either the RDT or thick blood smear were *P. falciparum* positive, the therapy recommended in 2004 was provided, consisting of oral chloroquine (10 mg/kg daily for 3 days) with sulphadoxine-pyrimethamine (1/2 tablet per 10 kg). After each clinical malaria episode, a follow-up finger prick blood sample for serum and thick smear was requested within 3 weeks of treatment.

### Malaria parasite detection and serum preparation

Thick blood smears and RDTs were performed directly from the finger prick samples. Giemsa-stained thick blood smears were read by two experienced microscopists examining 100 high-powered fields (×1000 magnification) to determine the presence or absence of detectable *P. falciparum*. Finger prick blood samples for antibody analysis were collected in serum separator tubes (BD Vacutainer systems, Oxford, UK) and stored on ice during transfer from the field to the laboratory. On arrival at the laboratory, tubes were centrifuged at 1100 ***g*** for 5 min, and sera removed for storage at −80°C prior to use in antibody assays.

### Parasite antigens

Recombinant proteins representing blood stage vaccine candidate antigens, AMA1, EBA175, MSP1_19_ and MSP2 (two allelic types) were used. The AMA1 antigen was the full-length ectodomain, expressed in *Escherichia coli*, representing amino acids 83–531 of the *P. falciparum* strain 3D7 AMA1 (17). The EBA175 protein was a baculovirus-expressed 8His-tagged antigen representing amino acids 144–753 of the EBA175 sequence in the 3D7 strain, corresponding to the cysteine-rich region II (18). Three *E. coli*-expressed GST fusion proteins were used, one based on the MSP1_19_ sequence of the Wellcome *P. falciparum* strain (19), and two based on MSP2 amino acids 1–184 and 22–247 of the CH150/9 (type A) and Dd2 (type B) alleles respectively (20). The GST fusion tag alone was also expressed from the pGEX-2T vector as a negative control antigen. *P. falciparum* parasites from the 3D7 parasite clone were cultured to prepare schizont extract as crude *P. falciparum* parasite antigen.

### Antibody assays

Serum IgG antibody reactivities were measured by ELISA absorbances (optical density, OD) at 490 nm, following previously described methods (21). Briefly, each well of 96-well plates (Immulon 4 HBX, Dynatech, Fisher Scientific, Loughborough, UK) was coated with 50 ng recombinant antigen, or 5 μg crude schizont extract antigen, in 100 μL coating buffer overnight at 4°C. Plate wells were washed four times in 0·005% Tween20 (PBS/T), and blocked for 4 h with 1% milk powder in PBS/T. Test sera were added in duplicate at 1/500 dilution (in PBS/T with 1% milk powder), and incubated overnight at 4°C.

To assess antibody avidity, chaotropic anion dissociation of antibody–antigen interactions with 100 μL guanidine thiocyanate at 4, 3, 2, 1, 0·5 and 0·1 m concentrations was used with a pool of positive control sera, collected from nine adults from the Brefet region of The Gambia, who had high ELISA OD antibody reactivities to all antigens (22). A single concentration of 0·5 m guanidine, in duplicate, was selected to examine relative antibody avidity for test serum to antigens. This concentration of guanidine was suitable to elute antibody within the log-linear phase of the curve for all antigens, according to positive control samples. Plates were incubated for 12–15 min at ambient temperature before removal of guanidine by washing in PBS/T. Wells were washed four times, then incubated for 3 h with 100 μL of horseradish peroxidase-conjugated goat anti-human IgG (Dako UK, Ltd, Cambridgeshire, UK) at 1/5000. After four further washes, 100 μL substrate solution was added (0·4 mg/mL o-Phenylenediamine dihydrochloride, 0·1 m citric acid, 0·2 m Na_2_HPO_4_), and incubated for 15 min before adding 50 μL 2 m sulphuric acid and determining OD at 490 nm. To generate relative antibody avidity indices for test sera, the ratio of ELISA OD for sera incubated in the presence of 0·5 m guanidine thiocyanate, and OD without guanidine incubation, were calculated for AMA1, MSP1_19_, MSP2A and MSP2B antigens.

### Statistical analysis

Estimates of the persistence of dry season antibody responses were made previously for children with samples at five or more time points, from day 0 until the end of the 12-week dry season study period. Dry season ELISA absorbance OD values were transformed into standardized units using a standard curve. For comparison with wet season ELISA ODs, persistence of dry season antibody response was determined by a single parameter: the lowest antibody level estimated during the dry season as a proportion of the antibody level at day 0 (10).

To examine differences in antibodies between groups, the two sample *t*-test for parametric data or the Wilcoxon rank sum (Mann–Whitney) test for nonparametric data (on non-normally distributed data or data with unequal variance between sample groups) was used. Linear regression was used to test associations between continuous dependent and independent variables. For normally distributed data, unadjusted Pearson’s *R*^2^ coefficient (slope between *x* and *y*) and *P* values are reported. For non-normally distributed data, Spearman’s Rho, and *P* values are reported. For children with clinical malaria, comparisons were made between proportions with and without antibody increases >0·5 OD ELISA units to individual antigens (0·5 ELISA OD represents a value greater than 3 SDs above the mean negative control values for each antigen and allows for comparisons between antigens).

## Results

### Follow-up of the cohort and clinical episodes

Written informed consent was given for 152 children to participate throughout the dry season study. Of this total, 138 participants completed the 12-week dry season survey, and 124 children provided samples at five or more time points from day 0 until the end of the dry season; these samples were analysed for persistence of antibody response (10). After appropriate additional informed consent, 118 of the children participated in the subsequent wet season study. By the end of the wet season, there were 103 children with samples available at three cross-sectional time points: early during the dry season in February (day 0 for each individual child), early in the wet season before malaria incidence had increased (mean day 154, range 143–179), and the end of the wet season (mean day 285, range 274–299). *P. falciparum* parasites were detected in blood smears from 49 (48%) of the children early during the dry season, from six (6%) of the children early during the wet season and from 30 (29%) of the children at the end of the wet season. In total, during the wet season, *P. falciparum* parasites were detected in blood smears of 46 (45%) of the children.

Thirty four (33%) of the children were determined to have experienced an episode of clinical malaria. These children had parasite positive thick smear or RDT with fever, or reported history of fever within the previous 48 h, and were treated for clinical infection. There were 21 children with an episode of clinical malaria from whom a blood sample was collected at the time of diagnosis and another sample collected within 18 days after the start of treatment for clinical infection, allowing antibody profiles to be examined in detail for these children (for the remaining 13 children with an episode of clinical malaria, samples were not collected post-treatment).

The mean age of children at the start of the dry season was 3·97 years (SD 1·53). There was no significant age difference between children who experienced an episode of clinical malaria during the wet season (mean 4·27 years, SD 1·53) and those who did not (mean 3·83 years, SD 1·52). The cohort was composed of 43 females and 60 males, and episodes of clinical malaria were not associated with gender (female *n*= 14, male *n*= 20).

### Antibody levels were boosted during the wet season

**Figure 1:**
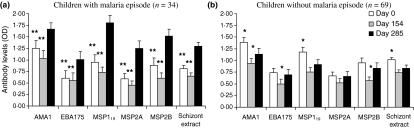
Changes in mean antibody levels between early dry season (day 0), start of malaria transmission season (mean day 154) and end of the malaria transmission season (mean day 285) for children with (a, *n*= 34) and without (b, *n*= 69) a recorded episode of clinical malaria during the transmission season. Significant differences between days 0 and 285 or days 154 and 285 antibody levels are shown (**P< 0·01, *P< 0·05).

Mean serum antibody levels to all of the parasite blood stage antigens increased during the wet season in children who experienced an episode of clinical malaria, being significantly higher at the end of season (day 285) than pre-season (days 0 and 154, Figure 1a). Among those who did not have an episode of clinical malaria, there was much less evidence of boosting in the group as a whole. Mean antibody levels at day 285 were lower than at day 0, and they were only marginally higher than at day 154 (Figure 1b). However, there were antibody increases greater than 0·5 ELISA OD units to one or more antigens in 24 (35%) of the children who did not have clinical malaria, as well as in all of the children who had clinical malaria.

Although this cohort study was not statistically powered to investigate whether antibody reactivities were associated with protection from malaria, children who experienced clinical malaria had lower initial (day 0) antibody levels (mean ELISA OD = 0·805) to *P. falciparum* schizont extract than children who remained free of clinical malaria (mean ELISA OD = 1·013; *P*= 0·029). The trend was similar for the individual recombinant antigens, although these were not significant (Table 1).

**Table 1:** Comparison between mean (SD) antibody optical density values at day 0 early in the dry season for children with and without clinical malaria during the wet season

Antigen	No clinical malaria (*n*= 69)	Clinical malaria (*n*= 34)	*P*-value
AMA1	1·39 (0·88)	1·24 (1·07)	0·48
EBA175	0·74 (0·81)	0·60 (0·92)	0·46
MSP1_19_	1·18 (0·83)	0·95 (0·93)	0·21
MSP2A	0·67 (0·70)	0·59 (0·71)	0·57
MSP2B	0·95 (0·86)	0·88 (0·92)	0·71
Schizont extract	1·01 (0·44)	0·81 (0·46)	0·03

### Associations between dry and wet season antibodies

Longer persistence of antibodies during the dry season was significantly associated with higher end of wet season (mean day 285) antibody levels for all antigens except MSP2 (Table 2). The data indicate that the elevated ability to sustain antibody levels can persist throughout the dry and wet seasons in young children. No differences in persistence of dry season antibody response, or dry season parasitaemia, were observed between children who had clinical malaria during the subsequent wet season and those who did not.

**Table 2:** Correlation co-efficient between dry season lowest antibody concentrations as determined previously (10) and antibody levels at the end of the following transmission season (mean day 285)

Antigen	*R*^2^	*P*-value
AMA1 (*n*= 77)	0·06	0·03
EBA175 (*n*= 26)	0·33	<0·01
MSP1_19_ (*n*= 69)	0·06	0·04
MSP2A (*n*= 42)	0·08	0·07
MSP2B (*n*= 60)	0·03	0·17
Schizont extract (*n*= 64)	0·08	0·03

### Antibody response profiles for children with clinical malaria

**Figure 2:**
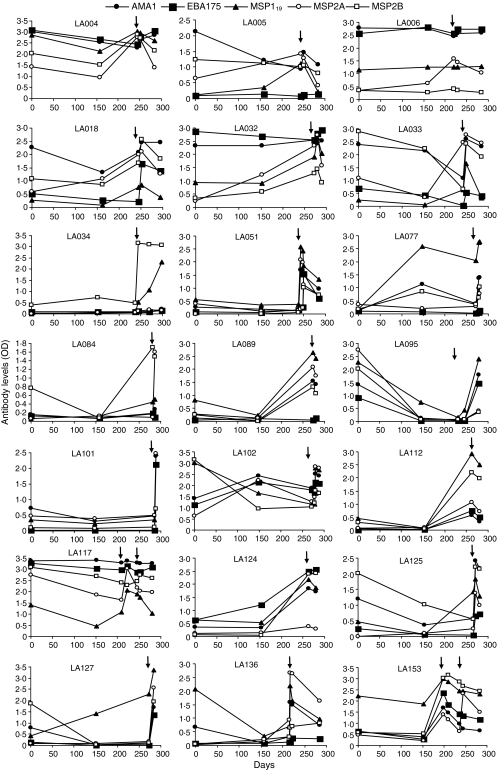
Longitudinal antibody profiles for 21 children with an episode of clinical *P. falciparum* malaria during the transmission season and a follow-up sample collected within 18 days post-treatment. An arrow indicates day of treatment for clinical malaria. Day 0 is early in the dry season (February), and day 154 is at the beginning of the wet season (July) after which time most malaria transmission occurs.

Very diverse longitudinal antibody profiles were observed in children who experienced clinical malaria. Individual antibody profiles are shown for 21 children who had clinical malaria during the wet season and from whom a follow-up sample was obtained within 18 days post-clinical episode (Figure 2). Some children maintained elevated specific antibody levels throughout the dry and wet seasons (LA004, LA102, LA117). Other children had low antibody levels throughout both seasons until the time of clinical malaria (LA051, LA101) or declining antibody levels during the dry season and elevated levels at the time of clinical malaria (LA095, LA136). Thus, children with highly diverse longitudinal antibody profiles were vulnerable to experience an episode of clinical malaria.

The individual profiles indicate that peak antibody levels usually occurred during or immediately following clinical infections. Such rapid responses co-incident with malaria episodes suggest robust immune memory. Antibody levels were often reduced from peak levels by the end of the wet season, but were generally higher than those observed at the beginning of the wet season. Some individuals (LA018, LA095, LA112, LA124) showed increases in antibody levels to all antigens tested, whereas others (LA034, LA101, LA125, LA89, LA112, LA124) showed increases in antibody levels only to particular antigens (Figure 2).

**Figure 3:**
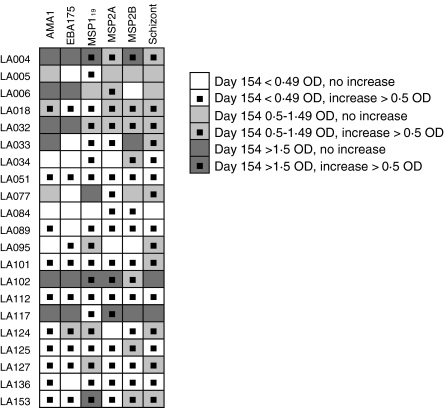
Summary of antibody level increases during the wet season (between mean days 154 and 285) in 21 children with a recorded episode of clinical malaria, stratified by antibody levels at the start of the wet season (day 154).

Figure 3 illustrates the occurrence of antibody increases of >0·5 ELISA OD units among these 21 children during the wet season. Throughout the wet season, antibody levels against EBA175 remained <0·5 ELISA OD units in seven (33%) of these 21 children, indicating limited boosting of responses to EBA175 compared with other antigens. Persistently low antibody levels of <0·5 ELISA OD were seen in only three children for AMA1 and MSP2A, in two children for MSP2B, and in only one child for MSP1_19_ and schizont extract (Figure 3). Despite the frequent low reactivity to individual antigens, all children with clinical malaria (*n*= 34) had increases of >0·5 ELISA OD in one or more of the antigens during the wet season.

### Lower antibody avidity measures for MSP2 antigens than AMA1 or MSP1_19_

The quality of antibody response was considered in terms of antibody avidity to individual recombinant antigens for samples with antibody levels >0·5 ELISA OD units. Avidities were neither examined for schizont extract, as this is a heterogeneous crude antigen preparation, nor for EBA175, as relatively few children had high antibody levels to this antigen.

**Figure 4:**
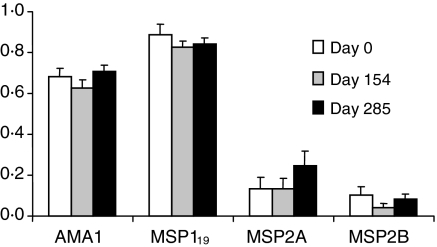
Antibody avidity measures for IgG to AMA1, MSP1_19_, MSP2A and MSP2B in serum from children with antibody levels >0·5 optical density (OD) at each time points. Relative antibody avidity indices (ELISA OD with/ELISA OD without 0·5 m guanidine thiocyanate incubation) during the dry season (day 0), beginning of malaria transmission season (day 154) and end of the malaria transmission season (day 285). AMA1 day 0: *n*= 74, day 154: *n*= 55, day 285: *n*= 70; MSP1_19_ day 0: *n*= 64, day 154: *n*= 47, day 285: *n*= 61; MSP2A day 0: *n*= 44, day 154: *n*= 32, day 285: *n*= 48; MSP2B day 0: *n*= 52, day154: *n*= 34, day 285: *n*= 56.

At all time points, lower antibody avidities were observed to both allelic forms of MSP2, compared with MSP1_19_ and AMA1 (Figure 4). The validity of exact comparisons between differing types of test antigen is uncertain, but highest avidity indices were seen to MSP1_19_ (∼0·8), followed by AMA1 (∼0·6) and avidity indices to MSP2 antigens were markedly lower (∼0·1 to 0·2) (Figure 4). Avidity indices for a positive control pool of nine sera from Gambian adults were higher (0·90 for AMA1, 0·94 for MSP1_19_, 0·65 for MSP2A and 0·54 for MSP2B). There were positive associations between antibody levels and antibody avidity index at all time points for AMA1 (day 0: *R*^2^ = 0·21, *P*< 0·001; day 154: *R*^2^ = 0·26, *P*< 0·001; day 285: *R*^2^ = 0·16, *P*= 0·001) and MSP1_19_ (day 0: *R*^2^ = 0·11, *P*= 0·007; day 154: *R*^2^ = 0·08, *P*= 0·048; day 285: *R*^2^ = 0·16, *P*= 0·001), but not for MSP2 antigens. There were no consistent associations between antibody avidity measures and age or persistence of antibody response. There were also no observed correlations between antibody avidity and presence of parasite infection, or occurrence of clinical malaria.

## Discussion

In this cohort study in The Gambia, increases in serum IgG antibody levels to *P. falciparum* merozoite antigens were observed in many children during the wet season when malaria transmission occurred. Rapid increases occurred around the time of clinical infection in all children who had a clinical malaria episode for at least one of the tested antigens, indicating memory responses that were boosted during the infections. Following treatment and resolution of clinical malaria, antibodies to specific antigens can commonly be detected for months, although the levels decline (7,8). A previous analysis of the dry season period in the Gambian cohort studied here showed that elevated antibody levels can be maintained for extended periods in some individuals even in the absence of persistent infection, particularly in those older than 5 years of age (10). Children with more persistent dry season antibody responses also had higher antibody levels at the end of the wet season, compared with children who had more rapid dry season antibody decline. Antibody levels to all antigens were higher at the end of the wet season in those who had clinical malaria infections, but these levels were sometimes lower than the peak levels observed around the time of infection. All children with clinical malaria had increases in antibody levels of one or more antigens by the end of the wet season, whereas such increases were only seen in approximately one-third of those who did not have a clinical malaria episode detected.

Rates of dry season antibody decline to *P. falciparum* antigens in the absence of persistent infection were previously associated with age of children and differences between malaria antigens (10). Antibody decline rates to Pneumococcal capsular polysaccharide vaccines have also varied among antigens tested (23). It is possible that the rate of antibody decline may correlate with protective efficacy, and although there was no association with risk of malaria in the present study, this could be investigated in a larger study.

Antibodies with low avidities are considered to be produced mainly as a result of non-germinal centre B cell development, but excessive stimulation of B cells and maintenance of large numbers of somatically mutated B cells could also be associated with low avidity antibodies in response to malaria antigens (24). Increasing antibody avidities have been described to MSP1 and schizont extract antigens following malaria infection (11,14), but recent data also suggest that malaria infection can inhibit affinity maturation of antibodies (25). Naturally acquired antibody avidities to malaria antigens have not been previously investigated among young children, and interestingly we found that these did not change between different sampling periods in relation to seasonal malaria transmission. Although high antibody levels were observed to MSP2, antibody avidities to this antigen were constantly low, suggesting that exposure to new infections did not boost avidities to comparable levels with those for AMA1 or MSP1_19_ in this population. There is evidence that MSP2 antigens promote an IgG3 skewed antibody response compared with other malaria antigens (26). While IgG3 antibodies have a shorter half life than IgG1 antibodies, the relative affinity of different antibody subclasses to malaria antigens has not been defined. A *P. chabaudi* murine vaccine study showed no differences between antibody avidities to AMA1 or MSP1 conformation-dependent and conformation-independent epitopes (27; MSP2 could not be compared as that antigen is not present in rodent malaria parasites). Less persistent antibody responses were observed to MSP2 than to AMA1, EBA175, MSP1_19_ and schizont extract antigens during the dry season amongst children in this cohort (10). Taken together, these observations suggest that MSP2 may promote less mature antibody responses than those to other antigens such as AMA1 and MSP1_19_. It is unknown whether the differences in MSP2 antibody avidities observed in this study relate to a particular structure of the MSP2 antigens within the tandem repeat sequences, or the polymorphic nature of some of these repeat sequences (28). Examination of antibody avidity in detail against reagents representing different parts of the MSP2 antigen would be necessary to further investigate this.
